# Participant Experiences with EMPOWER: Benefits, Barriers, and Best Practices for an Online Peer Mentorship and Leadership Program for Women in Academic Medicine

**DOI:** 10.1177/26884844251403443

**Published:** 2025-01-01

**Authors:** Rochelle D. Jones, Ying-Jen Lin, J. Denard Thomas, Audrey M. Blake, Nancy D. Spector, Christina M. Cutter, Kanakadurga Singer, Kelly C. Paradis, Eve A. Kerr, Eva L. Feldman, Abigail J. Stewart, Isis H. Settles, Peter A. Ubel, Reshma Jagsi

**Affiliations:** 1 Department of Radiation Oncology, Emory University School of Medicine, Atlanta, Georgia, USA.; 2 Center for History, Humanities, Arts, Social Sciences, and Ethics in Medicine (CHHASSEM), Michigan Medicine, University of Michigan, Ann Arbor, Michigan, USA.; 3 Department of Pediatrics, College of Medicine, Drexel University, Philadelphia, Pennsylvania, USA.; 4 Executive Leadership in Academic Medicine® (ELAM), College of Medicine, Drexel University, Philadelphia, Pennsylvania, USA.; 5 Department of Emergency Medicine, Michigan Medicine, University of Michigan, Ann Arbor, Michigan, USA.; 6 Department of Pediatrics, Michigan Medicine, University of Michigan, Ann Arbor, Michigan, USA.; 7 Department of Radiation Oncology, Michigan Medicine, University of Michigan, Ann Arbor, Michigan, USA.; 8 Department of Internal Medicine, Michigan Medicine, University of Michigan, Ann Arbor, Michigan, USA.; 9 VA Ann Arbor Healthcare System, Center for Clinical Management Research, Ann Arbor, Michigan, USA.; 10 Department of Neurology, Michigan Medicine, University of Michigan, Ann Arbor, Michigan, USA.; 11 Department of Psychology, University of Michigan, Ann Arbor, Michigan, USA.; 12 Department of Women’s and Gender Studies, University of Michigan, Ann Arbor, Michigan, USA.; 13 Fuqua School of Business, Sanford School of Public Policy, and Department of Medicine, Duke University, Durham, North Carolina, USA.

**Keywords:** leadership development, faculty development, peer mentoring, online learning, virtual mentoring

## Abstract

**Background:**

We developed the Engaging Peer Mentors for Opportunity, Well-Being, and Equity Realization (EMPOWER) program to provide leadership training and peer mentoring in a virtual, scalable format. Designed to be widely accessible to women leaders in academic medicine, it combined an asynchronous online curriculum with 1 hour per month group meetings *via* teleconference with peers and a faculty advisor. This qualitative study assessed the program’s feasibility and impact as well as identified areas for quality improvement.

**Methods:**

We conducted individual interviews with 34 program participants as well as focus groups with 14 faculty advisors. These were conducted virtually at the program midpoint and following program completion. The Framework Method informed qualitative analysis.

**Results:**

Beneficial program outcomes included knowledge acquisition/skill development, new or different ways of thinking, a sense of empowerment/self-confidence, and the clarification of personal values/goals. Monthly, 1-hour peer meetings appeared to further facilitate learning and practical application. Barriers included a lack of time to complete didactic activities and to attend group meetings, limitations of an online/virtual format, individual circumstances, and idiosyncratic group dynamics. Recommendations to improve quality and establish best practices included clear communication of well-defined aims/expectations, tailored programming, efficient use of time, minimization of labor/mental load, and enhanced online community-building.

**Conclusions:**

The benefits program participants and their faculty advisors described reinforce the value of gender-aware leadership development programs, especially those that include peer mentoring. By enhancing accessibility, engagement, and flexibility, programs such as EMPOWER can become more inclusive and effective, ensuring that all promising leaders can thrive in academic medicine.

## Introduction

Gender disparities persist in academic medicine. Although the number of women holding leadership roles has grown, structural barriers remain, including persistent salary disparities,^1^ sexual harassment,^2^ gendered stereotype threats,^3^ and more limited advancement opportunities for women compared with their male counterparts. These challenges hinder women’s career progression and perpetuate the underrepresentation of women in all leadership roles,^3–5^ which in turn, makes it unlikely for institutions to cultivate an inclusive and supportive culture for women in academic medicine.^5^

Organizations advocating for women in science have identified mentorship, sponsorship, and leadership support as key priorities to address the challenges women continue to face in academic medicine, helping them navigate the complexities of career advancement.^4^ Providing structural leadership development programs and effective mentorship for women can potentially and significantly enhance job satisfaction, career advancement, and productivity.^6–8^ However, one of the persistent barriers to women’s career progression in academic medicine is the lack of senior women mentors who can serve as role models.^7,9^ In response, peer mentoring^10–12^ has emerged as an alternative model that provides both career and socioemotional support.

When structured thoughtfully and implemented within a safe and supportive environment, peer mentoring can help women build the confidence, networks, and strategies to overcome gendered challenges.^11^ Beyond mentorship, sponsorship^3^ is particularly crucial for advancing women’s careers, as it increases visibility and facilitates access to leadership opportunities. The Hedwig van Ameringen Executive Leadership in Academic Medicine (ELAM®) program is a well-established national initiative to address the need for sponsorship and has played a significant role in advancing gender equity in academic medicine. ELAM® ^8,13^ offers an in-depth, experiential curriculum that focuses on essential leadership competencies while incorporating facilitated peer mentoring to promote reflection, collaboration, and inspiration. The program has led to notable successes, with alumnae securing high-profile leadership roles, including dean-level positions, and increased research productivity through grants and publications. However, despite its effectiveness, ELAM® has limitations, including restricted scalability and limited reach, as each institution can nominate only a small number of participants; further, the program requires frequent in-person participation, which may not be feasible for all candidates.

Recognizing the need for a more accessible and scalable solution, we developed the Engaging Peer Mentors for Opportunity, Well-Being, and Equity Realization (EMPOWER) program,^14^ drawing inspiration from ELAM® while adapting its principles to a fully virtual format. EMPOWER aims to provide fundamental leadership training and facilitated peer mentoring in a way that is more widely accessible to women in academic medicine. A detailed description of the year-long EMPOWER program’s structure and curricular content is reported elsewhere.^14^ In brief, the key learning areas EMPOWER addresses are leadership effectiveness, negotiation, strategic career advancement (including sponsorship and self-promotion), and work–life integration. The curriculum features content specifically tailored toward the experiences of women faculty in academic medicine. This includes readings and media presenting on issues related to gender, culture, bias, hierarchical structures, and/or power dynamics that might influence how a woman establishes her leadership style,^15–17^ approaches negotiations,^18,19^ or strategizes career networking, sponsorship, and self-promotion,^20,21^ for example.

An invitation to participate in a pilot of the EMPOWER program was extended to a cohort of women medical faculty in academic positions who had received National Institutes of Health K08 or K23 career development awards from 2006 to 2009, as identified through our prior survey studies.^2^ Program participants accessed curricular materials through an online learning platform and met virtually for 1 hour per month with their peer mentorship circles to engage in discussion and collective learning. Each circle was composed of up to six peer members as well as a faculty advisor who facilitated and served as a source of senior guidance and support. Peer circles were composed of members in the same or adjacent time zones, but from different institutions and varying specialties, to facilitate scheduling and ensure confidentiality, respectively. All peer members and their faculty advisors were women, and all faculty advisors were recruited from a list of alumni of the ELAM® program. The peer circle was meant as a forum in which participants could further engage with their peers and faculty advisor on the curricular content as well as topics that are particularly salient to them, including the gendered leadership barriers they might currently have been facing. Moreover, the group discussions and interactive exercises facilitated the sharing of new ideas and differential experiences among peers, allowing for the opportunity to challenge dominant paradigms within academic medicine and their own institutions as well as brainstorm alternative approaches to what constitutes leadership, worthwhile aspirations, and success.

In this qualitative study, we evaluated perceptions of and experiences with EMPOWER by conducting in-depth interviews with participants and their faculty advisors at both program midpoint and after program completion to assess implementation challenges and troubleshoot problems in real time, as well as capture final reflections. By examining their insights, we assessed the EMPOWER program’s feasibility and impact as well as identified areas for quality improvement to optimize this leadership development intervention for women in academic medicine.

## Methods

The Institutional Review Board of the University of Michigan Medical School approved this qualitative assessment as part of a randomized trial to evaluate the impact of the EMPOWER intervention.^14^ All participants were provided with the required informed consent information and agreed to take part in the study. Documentation of informed consent was waived because the research was considered no more than minimal risk. This qualitative study gained insights into EMPOWER’s feasibility, optimization, and scalability through in-depth, semi-structured, one-on-one interviews with program participants as well as focus groups with their peer circle faculty advisors.

Interviews and focus group guides were developed to assess experiences with and impressions of the program, including perceived benefits and barriers to participation as well as thoughts on how to optimize program implementation. Interviews and focus groups were conducted at the program midpoint (February–May 2023) and after completion (November 2023–February 2024), virtually *via* teleconference by two senior analysts (R.D.J. and Y.L.). The audio recordings were transcribed by professional transcriptionists.

Interviews were conducted with a sample of 34 out of the 89 program participants. Using a purposeful sampling strategy to maximize the diversity and range of viewpoints (*e.g.,* racial identity and program engagement),^22,23^ a participant from each of the 16 peer circles was selected for an interview at each time point. If there was no response to an invitation after sending a reminder, another participant was selected. In total, 54 individuals were invited to interview. Of the 54 invited, 20 did not accept the invitation, whereas 34 accepted and completed the interview. Each interview participant only received one invitation and completed one interview at either the midpoint or end of the program, resulting in at least one person per peer circle at each time point (17 at the mid-point and 17 at the end). Interviewee characteristics are presented in Table 1. The length of the interviews ranged from 20 to 60 minutes, with an average length of 37 minutes.

**Table 1.** Participant Characteristics

Demographics for qualitative interview participant sample (*N* = 34)		
	
Variable	Level	*N* (%) or mean (SD)
Gender^a^	Woman	34 (100)
Race^b^	White	19 (56)
	Asian	12 (35)
	URM^c^	3 (9)
Sexual orientation^a^	Heterosexual/Straight	34 (100)
Degree at K-award^b^	MD	20 (59)
	MD/PhD	5 (15)
	Non-MD	9 (26)
K-award grant type^d^	K08	10 (29)
	K23	24 (71)
K-award year^d^	2006	7 (21)
	2007	10 (29)
	2008	8 (24)
	2009	9 (26)
Specialty at K-award^e^	Basic sciences/non-MD	9 (26)
	Clinical specialties for women, children, and families	10 (29)
	Hospital-based specialties	1 (3)
	Medical specialties	12 (35)
	Surgical specialties	2 (6)
Region at K-award^f^	West	12 (35)
	Midwest	4 (12)
	Northeast	16 (47)
	South	2 (6)
Weekly work hours^a^	Continuous:	57 (11)
Weekly hours on caregiving and domestic tasks^a^	Continuous:	35 (22)
Weekly hours in patient care^a^	Continuous:	11.46 (11)
Marital status/partner employment status^a^	Single/divorced/widowed	2 (6)
	Married or domestic partnered, spouse or partner not employed or part-time employed	7 (21)
	Married or domestic partnered, spouse or partner employed full-time	25 (74)
Have any children^a^	Yes	34 (100)
Children requiring adult supervision^a^	Yes	19 (56)
Other adult dependent/s^a^	Yes	9 (26)
Academic rank^a^	Professor	12 (35)
	Associate professor	21 (62)
	Assistant professor/other	1 (3)
Endowed professorship^a,g^	Yes	6 (18)
Any institutional leadership position^a,h^	Yes	13 (38)
Any national leadership position^a,i^	Yes	23 (68)

a Self-reported from a survey conducted between 2021 and 2022.^2^

b Self-reported from a survey conducted between 2010 and 2011.^1^

c URM (underrepresented race and ethnicity in medicine) included 3 individuals who indicated they were Hispanic/Latino.

d Determined by searches of the NIH RePORTER database conducted in 2010.^1^

e Self-reported from a survey conducted between 2010 and 2011 and then categorized by the research team as described elsewhere.^1^

f Determined by internet searches of institutional websites conducted in 2010 to determine location and then categorized by the research team as described elsewhere.^1^

g Endowed professorship defined as a position that receives funds to support it from an endowment fund specifically designated for that purpose (may be synonymous with a distinguished professorship; does not include when a faculty member at a public institution draws FTE from the state of the institution).

h Institutional leadership positions (not mutually exclusive) included: *N* = 5 (14.7%) center directors; *N* = 4 (11.8%) fellowship or residency directors; *N* = 6 (17.6%) section or division chiefs.

i National leadership positions (not mutually exclusive) included: *N* = 12 (35.3%) member of board of directors or officer of a large professional society; *N* = 16 (47.1%) journal editor or editorial board member; *N* = 9 (26.5%) national committee or grant review study section chair.

SD, standard deviation.

All 16 peer circle faculty advisors were invited to participate in the focus groups. Those who responded to the invitation were assigned to a focus group based on overlapping schedules and availability. Seven focus groups were convened in total, with 14 out of the 16 faculty advisors participating in at least one focus group and with 9 able to participate at both time points. The length of the focus groups ranged from 37 to 57 minutes, with an average length of 50 minutes.

The Framework Method informed qualitative analysis of the transcripts.^24^ The first analysis phase involved an initial review of the transcripts and multiple iterations of inductive, open coding to allow unexpected concepts to emerge. During this process, 21 transcripts (14 one-on-one interviews and all 7 focus groups) were independently coded by at least two members of the research team. The team would then regularly meet to compare codes, resolve discrepancies, and reach consensus on definitions. The agreed-upon codes were then organized into categories, resulting in a codebook reflecting a working analytical framework. All transcripts were then divided between the two senior analysts (R.D.J. and Y.L.), who each systematically applied the working analytical framework using MAXQDA,^25^ a qualitative data analysis software. After all coding was complete, the applied codes from each transcript were further reviewed by a different analyst and summarized in grid tables with associated excerpts for additional review. Thematic saturation^26,27^ was reached when no new codes or new themes emerged, indicating the breadth and depth of insights had been captured. Throughout the analytic process, the research team repeatedly discussed and reevaluated their impressions and interpretations of the data until consensus was reached on finalized themes.

## Results

We present the results of our qualitative analysis, organized into four themes: (1) impressions surrounding EMPOWER program components and the leadership development process/learning cycle, (2) beneficial EMPOWER program learning and professional development outcomes, (3) barriers to EMPOWER program participation, engagement, and commitment, and (4) recommendations to improve EMPOWER program quality and best practices. Exemplar quotes are presented either within the text or in Tables 2 and 3 (denoted in the text as Q and numbered sequentially).

**Table 2.** Perceived Beneficial Outcomes in EMPOWER Program Learning and Professional Development

Outcome	Exemplar quotes
Knowledge acquisition/skill development	When I got the books, way before we even started meeting, I read [the negotiation book] immediately. I loved it. It was different than other negotiation thoughts that I’ve used myself which—I certainly still really value what I have from before—but I liked some of the added concepts and strategies that they suggested. That was fantastic, and I even used it when I was doing a negotiation before we started our classes or our circle groups. It worked amazingly. I was like, “Wow, this is great.” Yeah. That was good. (Participant, Q1) [EMPOWER] provided a framework of things to read about. I just think about some of the challenges that I faced in my academic career…I didn’t know how to phrase the questions to go ahead and find the material to answer it… I just wasn’t able to frame the question….it’s set up in such a way that it goes through this process of development. You know what I mean? Obviously, the [learning modules] are set up like that for a reason…putting them sequentially… that’s not something independently on my own I would’ve just been able to do. (Participant, Q2) There’s been some really good discussion about how some of the peers have negotiated for things at their institution and strategy. There’s this one peer member that has a lot of experience, and she even shared some books she’s read on the topic. She’s had a lot of training, so that was good. She’s been sharing books, and she’s been sharing strategies and experiences, so I’ve gained a lot, especially related to negotiation. (Participant, Q3) I think some of the ideas on how to prioritize your life and negotiating stuff was there, but never—they always seemed like abstract that, “oh, can I really put these into use?” …You could read something in a book about how you’re supposed to take care of a patient, but it’s very different when you have the patient in front of you, and you’re dealing with them. I think it’s the same where I can read about imposter syndrome… talking to people about it and how they’ve overcome it and how they implement some of these things, I think was very, very useful. (Participant, Q4) I think hearing personal stories from others and how they are thinking about their situations and how their responses have been—or their actions and behaviors have been responded to by their departments or whoever their leadership is—getting that insight into what’s going on with their professional careers has been really helpful for me. I think that’s something that, I feel like, is unique to women. The way we present ourselves and behave to the world is perceived and reacted to differently…I don’t think there’s enough information available, to me, for me to know how to best navigate the system. Getting a little bit more of this, personal narratives and information, from my peers is really helpful for me. (Participant, Q5)
New/different ways of thinking	It’s like the set aside time to think about the leadership constructs, and then again, sort through or grapple with or analyze what’s my style, what resonates, and then having the chance to think about, what does this apply to for me right now…it’s been a really good playground to be like, how do I engage people? How do I get buy-in? How do I lead this group? I think thinking through those pieces over time, while I’m working on [my leadership project for EMPOWER] has been helpful. (Participant, Q6) I thought [EMPOWER] was wonderful, and it let me think about things differently. That is what I appreciated a lot. It gave me a perspective on my career…it encouraged me to kind of take [an] observer-perspective on what it is I’m doing here. It pulled me out of the weeds, so to speak, and had me thinking about how to approach things with intention. I really benefited from that. (Participant, Q7) I also appreciated some of the exercises… Gaining some sort of hands-on practice to test out some of these new ways of thinking. It challenged some of my assumptions…yeah, I feel like it made me look at old problems in some new ways. (Participant, Q8) I think I would highlight the peer-to-peer nature of [EMPOWER] …even though the details of all our situations were so very different, there was such a common thread in what we were experiencing…The exposure to novel ways of thinking about the same things. I think we’re all aware that female faculty are less represented in leadership. I think we’re all aware that we get paid less and all these things, but then, it’s like, “Okay. How are some ways that we can think about that differently?” That was helpful. (Participant, Q9) I think it’s given me other ways to think about leadership and women in leadership and helped me see how I fit in, either the same or different from other women leaders who are going through some of the same things. (Participant, Q10) I think there was tremendous benefit for the opportunity to have career advancement conversations with individuals outside their home institution as a safe space to think big, to be challenged by others, to have their perspectives aired, and to learn new perspectives. I think that’s probably, to me, one of the greatest benefits is having that, building that community, that bigger community who really can help you have that environment to have those thought processes happen. I think that’s what I saw my individuals in my [peer circle] group really help each other, they kinda coached each other often to think in new ways, to challenge assumptions of what they could or couldn’t do. I think it happens differently when it’s a different institutional perspective, and they can bring their shared experiences to help another person grow. I think that’s a real highlight of the [EMPOWER] program. (Faculty Advisor, Q11) I think the most powerful piece…in our [peer circle] group, was the ability to develop strong ties with colleagues in different settings. It’s not just within your specialty, and it’s certainly not within your academic medical center, which I think offers a degree of freedom to discuss very sensitive issues and get thoughtful, as much as you can, unbiased responses from individuals to help guide and help you shape your thinking and decision making…I think that was really one of the greatest benefits of what I saw happen in the group. (Faculty Advisor, Q12)
Empowerment/self-confidence	[The EMPOWER program is] working to empower the person to influence the environment and create something, whatever is justified or based on expertise and data, et cetera. I think it’s a very good program. (Participant, Q13) I’ve taken leadership classes and programs before, and they always left me with the impression that to be a leader you had to be assertive, extroverted, basically more male like. I feel like [EMPOWER is] the first program I’ve been a part of that’s really emphasized leadership skills that come more naturally to me. Things like listening and relying on a team and just a much more collaborative style of leadership. The content has really resonated with me and helped to formalize a lot of things that I was already inclined to do, but maybe gave me a little more confidence that you can lead with these traits rather than having to be super aggressive all the time. (Participant, Q14) EMPOWER has played a critical role in helping me, largely in validating my feelings about things and certainly in helping me with strategies. For instance, that negotiation sheet. I pulled it out, and I will be using it at 10:30 with my meeting with my department chair. (Participant, Q15) [The EMPOWER program] had a very big impact because I was at a point of my career feeling like is this it? What else is there? …there are roadblocks…I think this helped empower me to say, “Listen, you have a solid career. You have a solid track record. You need to stand up for yourself and for what you believe and be more vocal.”… I think that has been very rewarding to have that backup from the [peer circle] group… Having that ability to discuss openly with a group that really they just want what they see the best for you…I think that was very, very rich and very empowering. Empower is the perfect name for it. (Participant, Q16) I feel like this group gave me some fortitude to not back down from what I’m asking, but also, maybe to think about some different ways to ask what I’m asking for or maybe not to react the way I’ve been historically reacting when I get bad news or when I encounter a barrier. It was just helpful to talk through some of my personal challenges. (Participant, Q17) I’ve gotten the confidence to reach out to say other institutions and kind of put myself out there, things that I would normally not do…the entire group really did empower me to take that extra step, to put myself out in more the national, international field. (Participant, Q18)
Clarification of values and goals	I think some of the book about looking at the different parts of your life and prioritizing…It was talking more like where you get disgruntled and burnt out is when what you want doesn’t align with what you’re doing… I really liked that concept and have moved that forward and used that…I think it’s given me —allowed me to give myself— a little bit of a break, like in a mental break and guilt break… I am looking at my career, and I see someone else doing this. That’s what they want, not necessarily what I want for myself. That’s okay. (Participant, Q19) One of the [assignments] that we had to do was, basically, set a goal for a change that we wanted to make…I met with a leader of our university, the provost of our university…What came out of that conversation was a leadership opportunity… she said, “You know what? Why don’t you apply for this?” …She suggested that I apply…so that I could kind of make strategic decisions… have a broader effect in an area that frustrated me. I went for that, and I got it. That was really exciting. I would say that some of the underpinnings that I was working on in the EMPOWER program really led me to grasp that opportunity. I heard it differently than I think I would have heard it had I not been in the program. I recognized it as, “Okay. She’s telling me to go for a leadership opportunity, and I should take it.” It also allowed me to say, “I’ve kind of been beating my head against a wall…and haven’t gotten anywhere, so maybe I just need to step off of that…”…That’s what I did, basically. I said, “I’m not doing this anymore. I’m doing this instead.” It felt very intentional. I felt like some of that intentionality came from having thought about it through EMPOWER. (Participant, Q20) I think it’s the practical piece…where do you want to go with your career? What are you gonna focus on? …Just getting other viewpoints on how to think about this and how to make these decisions…Those are the pieces I think that I find most useful so far…Do I go this path or this path? I’ve mentioned that in our group…It’s been nice to just think about that a little bit through this. (Participant, Q21) My own perspective was I didn’t think until recently that I was even cut out for leadership. That perspective has been similar among all of the members of the group…Do I really wanna get into this? That kinda thing. That perspective has been really valuable, to have other investigators who are really at very similar points in their careers and thinking similar[ly]. I imagined that if I were among a group of men, they might all be like, “well, of course, I wanna be a leader,” but to be in a group where it’s okay to discuss that—you might have some reluctance or questions or uncertainties about it—has been really nice. (Participant, Q22) One of the people in the [peer circle] group who had been aspiring or tapped to look at a higher-level position said, “…after going through this—all this process and thinking about everything more deeply, I realized I don’t want to just be moving up for the sake of moving up. I actually like what I’m doing now. I want to take this position I’m in and make this bigger and better and do things here and not just go to another position.” I think they all found it useful in some way, but they really learned from each other. (Faculty Advisor, Q23)

EMPOWER, Engaging Peer Mentors for Opportunity, Well-Being, and Equity Realization.

**Table 3.** Perceived Barriers to EMPOWER Program Participation, Engagement, and Commitment

Barrier	Exemplar quote
Lack of time and limited availability/scheduling issues	I think sometimes the reading assignments are hard, right, in terms of, if we have to pre-read—I mean, in an ideal world, I would love to, but I think I’m delinquent…I’ve been delinquent on some of them, but some I have had time to read beforehand. I don’t know that that’s a bad thing necessarily. It’s just kind of the reality of the schedule…I think it’s just intentionally finding the time to do it…. I just, for whatever reason, have found it, in some instances, to be challenging. (Participant, Q24) That’s been very difficult because people are just so stressed and busy. It hasn’t prevented them from participating [in the peer circle meetings]. Some of them actually were doing [the homework] as we were going through the session, which we’re all adults. It’s fine. (Faculty Advisor, Q25) It’s hard to connect in an hour… it would probably be good to have maybe an hour and a half or two hours to be able to discuss and share a little more deeply for each person.… running right up onto the hour…definitely more than once, [our faculty advisor] has suggested—and if we need to, we can try to schedule another time. As you know, it is a miracle to find a time that all the people can get together. (Participant, Q26) I think our circle ended up with a five o’clock meeting. I know it’s almost impossible to find a work hours meeting that everyone can go to…five to six is borderline after hours, but we’re definitely cutting into the personal time a little bit more than the work time to get it done…. I would prefer [this type of program be done during work hours], but I do realize that that would be extremely difficult to schedule, because of everybody’s work time schedule. (Participant, Q27) I feel that [the EMPOWER program] was a very positive experience for me as a faculty mentor advisor. I think it was also frustrating at times in terms of some of the scheduling things and issues with that… (Faculty Advisor, Q28)
Limitations of online/virtual format	It was really hard to get access to the materials. Not that you guys didn’t make it as easy as humanly possible, but having to log in to another [online learning platform], and remember my login account, and remember to do it and download it—there was something that we were supposed to do every month that was an online journal. I think I did it the first month, and I never did it again… (Participant, Q29) I have a few people in the [peer circle] group who I think always are diligent about going into [the online learning platform] …They’ve clearly watched the video and read the article… they’ve really done some prep work. I think I have others who, for whatever reason, have decided that getting into [the online learning platform] is just impossible…It’s not hard, but they’ve just decided that there’s gonna be a block on having another password or another thing…and that influences their participation. (Faculty Advisor, Q30) I think the one downside I can see is, it’s been hard to establish a group dynamic remotely, when the only time we talk to the other people in our circle are in these really structured Zooms…I think you miss out on the hallway conversations, coffee conversations that you would have in an in-person seminar. (Participant, Q31) One of the hard parts about Zoom is you don’t really see full body language, so it’s hard to know who’s gonna speak, and people speaking over one another and giving folks the space to speak without interruption. (Faculty Advisor, Q32) I guess I thought when I started [EMPOWER] that the level of participation would be more…because we weren’t all present together, it became easier to be like, “oh, I didn’t do the prep.” It feels like there was a little bit less accountability, because we were just on Zoom together…there were some assignments where we were supposed to write down [a plan], and I didn’t always do that…nobody was looking for me to come with a piece of paper…I’m sorry to admit this, but I didn’t always do my homework, and I think, “would I have gotten more out of it if I did all that? Yes, probably.” …Even if I don’t actually write it all down, I’m thinking about it, so I’m getting something out of it. I’m not getting the full experience. (Participant, Q33)
Individual circumstances	My kids are grown up now, I don’t have to worry about childcare—I think for those who are younger in my peer [circle] group that has been a little bit of an issue in terms of finding the time, I believe, so people usually have a spouse or a partner taking care of their children when they are attending these calls. (Participant, Q34) One of the things that we did when sensitive things came up [during a peer circle meeting], and it was taking more time… if it looked like this was something really important and we shouldn’t cut it off… saying, “I think this is a really an important discussion. I can stay on longer. What would you like to do?” Because one of the hard parts of increasing it to an hour and a half is a lot of folks—they’re balancing kids. An hour and a half may seem overwhelming…but in the moment, when everybody’s really intensely in it, intently in it, there’s more flexibility. Those who couldn’t do it would drop off, but those who could were able to find a way. (Faculty Advisor, Q35) I’m personally in a point in my career and a point in my professional experience and knowledgebase… some of the topics in those [curricular] materials are just less relevant. I think I might be a bit further along in my career than some of the other participants… I think for me, some of the drop-off in engagement happened for me personally, just because of where I am in my career, and I’ve come to understand some of the topics or issues already and that contributed to a fall-off in engagement, at least with the materials. (Participant, Q36) I think when I started with this [EMPOWER] program… I was looking for a leadership course because I was moving into a leadership position…some of the things that, again, were more practical of how to be a leader, I think were things that I used. Like the negotiating thing, I was actually negotiating for new hires and things like that at the time. I think professionally, I was able to use the things that were more leadership-focused. I think the things that were more professional development within myself, like getting my career to where I want it to go, I have not used just because that is not what I need right now. Right now, I’m trying to settle into a leadership role more so than advance myself in different ways. (Participant, Q37) Just because of just timing of my own circumstances, it would’ve been great to start [EMPOWER] like six months earlier. Yeah. I had some fairly big disagreements with my Chair, and so I’ll be leaving my department. Some of those negotiations happened at the tail beginning of it all. Maybe it would’ve been nice to have, like I said, the EMPOWER [program] earlier, but who’s to know? … [EMPOWER] came at a time that was, like I said, it would’ve been much more relevant six months earlier. (Participant, Q38)
Group dynamics	I think that was one of the hard things, is not everybody was there all the time, and so it was hard to feel like that level of familiarity with people to just have a conversation if people weren’t there all the time… I found [it] a little bit hard to have conversations with the group on some of the topics if people hadn’t had the time to like read the stuff or do the stuff. For example, there were some assignments that I thought were super interesting, and I would’ve been really excited to talk more about, but not everybody would’ve read them, so it was hard to have that conversation. (Participant, Q39) Our group is all white women… there’s maybe some religious diversity. There’s not a lot of diversity in race and ethnicity. Depending on the year, 30 to 50 percent of my mentees are people from populations underrepresented in medicine. I struggle with how to mentor them and to know the issues. Obviously, I ask them, but I think some perspective on that in our group would be nice. (Participant, Q40) I wonder how you mixed [the peer circles] together ‘cause I think my group [has not] come together that well. They’re nice people again, nice people, but I think it’s really been hard for us to—at least for me to — feel like these are people I have anything really in common with… I think the big question is what characteristics are most important to bring people together in peer groups… I don’t know if it would be something more related to where they are in their career or research interests or what kind of work/life balance issues… Especially in my group, there’s been some difference in training. (Participant, Q41) I personally felt a tension between being a [learning community advisor] vs. a teacher in this setting and my role in vertical mentoring vs. just facilitating the sessions was a little bit trickier for me to navigate. (Faculty Advisor, Q42) We did have one participant who tended to monopolize the time.…our faculty advisor would try to help her problem-solve or try to normalize what she was going through or validate. That only served to increase the amount of talking that she was doing… I think that that impeded our kind of coming together… I think that’s a real challenge in group dynamics in any kind of setting. (Participant, Q43) I mentioned a few things about [negative experiences in my career] as an example of the topic we were discussing and thinking that people would either ask me more about it or share a negative experience… That really didn’t happen, and I think the facilitator contributed to that not happening. Definitely, the facilitator needs to make sure we’re not off track, and we’re staying a positive discussion and we’re not hanging on negative past experiences. I agree with that…But I think it was a little bit out of balance, in that sharing negative experiences, sharing either personal growth or professional growth that came from them, opportunities that came from them, this group did not tend to focus on that at all… Part of it was, I think, the facilitator shifted us away from negative topics a little sooner than might have benefitted the group… (Participant, Q44) On the one hand you want the [faculty advisor] to let the group decide how they wanna do things, but I think it is also helpful when you have a bunch of people who don’t really know each other [to have] someone take the lead…we’re here because we’re trying to become better leaders, but… the group felt a little leaderless…. our [faculty advisor] was trying to let us self-direct… if no one’s saying anything, I don’t wanna be the one who’s always talking… we needed a little bit more direction, but I’m not sure where that should have come from. (Participant, Q45)

### Impressions of EMPOWER program components and the leadership development process/learning cycle

**FIG. 1.**
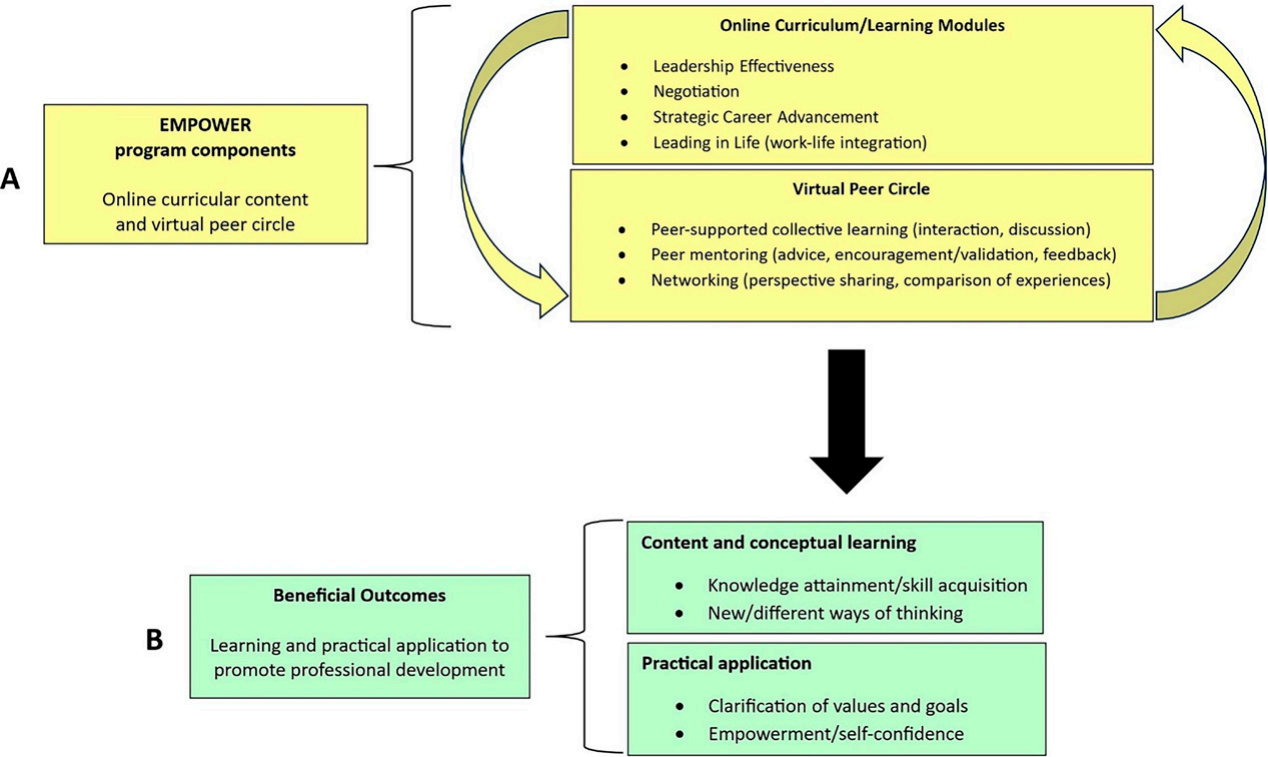
EMPOWER learning cycle and perceived beneficial outcomes. The EMPOWER leadership development process and learning cycle involve the reinforcement of the online curriculum through virtual peer circle interactions **(A).** Perceived beneficial learning and professional development outcomes of the program include content and conceptual learning as well as practical application at home institutions, bolstered by the clarification of personal values/goals and as well as the cultivation of leadership empowerment/self-confidence **(B)**. EMPOWER, Engaging Peer Mentors for Opportunity, Well-Being, and Equity Realization.

Many participants found the online curriculum informative and perceived that the virtual peer circle further facilitated learning and application of knowledge. Peer circle interactions were seen as opportunities for further review of the curricular subject matter as well as additional discussions on salient topics related to career and work–life experiences that, while extending beyond the intended curriculum, were important to participants. These peer exchanges were often described as including elements of reflection, problem-solving, experience sharing, and gathering of feedback as well as receipt of social support and encouragement. This characterization is consistent with the EMPOWER leadership development process and learning cycle, which is the reinforcement of the online curriculum through virtual peer circle interactions (Fig. 1A).

I thought it was—throughout the entire program—it was excellent. I really felt like it was a safe space. They were amazing, inspiring women. I learned a lot from them in how we tried out the material, how we reported back on things, other struggles that had tangential relationship to the EMPOWER curriculum….I just really felt like everyone went out of their way to provide support, and suggestions, and help, and empathy to all of the members of the peer mentoring group. (Participant)

I think that over the course of the year, I think we really helped one another….[The EMPOWER participants] were really incredibly thoughtful in how they helped one another address many of the challenges that they were encountering. They were able to tie the homeworks and the readings into what they were going through and how they might use it. I thought that was actually incredibly powerful. (Faculty Advisor)

### Beneficial EMPOWER program learning and professional development outcomes

Perceived beneficial program outcomes were organized into two types of learning and professional development (Fig. 1B). The first reflects content and conceptual learning, including knowledge acquisition, skill development, and new or different ways of thinking. The second involves the practical application of the acquired knowledge, skills, and thought processes to real-life situations at participants’ home institutions, such as exerting influence, making decisions, or pursuing career goals and milestones. This practical application was often associated with the development of participants’ sense of empowerment and self-confidence, as well as the clarification of their values and goals.

#### Knowledge acquisition/skill development

Many participants described the EMPOWER learning modules as featuring new or alternative information (especially exploration of subject matter that they might not have pursued on their own), structured content that complements, supplements, or reinforces prior learning, and a curricular framework featuring thoughtfully curated content that can be practically applied (Table 2; Q1, Q2).

In addition, many participants noted that the peer circle further increased the extent of their knowledge acquisition and skill development by providing them the opportunity to review curricular topics and practice skills with peers in an interactive setting (Q3, Q4). The discussions with peers also helped contextualize the subject matter in ways that reflected their real-life experiences, especially the challenges unique to women in academic medicine (Q4, Q5). Several participants reported receiving knowledge and feedback, as well as practical suggestions and actionable strategies, for navigating career challenges based on their peers’ experiences, viewpoints, and perspectives beyond their local institution (Q3, Q5).

#### Different ways of thinking

Participants often commented that they benefited from curricular materials and exercises that introduced them to new or different ways of thinking conceptually about their careers. These included alternative understandings of leadership, a broader perspective when thinking about one’s career path, deeper self-reflection on professional development, and different approaches toward dealing with issues and problem-solving (Q6, Q7, Q8).

Participants reported that the opportunity to meet other women leaders in their peer circle further solidified an alternative outlook on women in leadership (Q9, Q10). Some found that the peer circle discussions often stimulated brainstorming surrounding novel approaches to commonly held challenges in academic medicine and/or facilitated more objective self-reflection (Q9, Q10). Faculty advisors observed that participants were able to compare how commonly held issues were handled at different institutions and gain a wider perspective based on multiple seemingly unbiased viewpoints (Q11, Q12).

#### Empowerment/self-confidence

Participants reported feeling a sense of empowerment and self-assurance backed by their newly acquired knowledge, skills, and thought processes (Q13). They described improved morale and feeling validated as well as increased leadership self-confidence, especially in terms of being women leaders (Q14, Q15). Some detailed feeling further empowered to start implementing their skills in real-life situations to exert influence and/or pursue action at their own institutions (Q15).

Participants reported receiving social support and validation from their peers when they struggled with self-doubt or external barriers, which would help them persist and advance (Q16). The peer circle also provided a forum where participants could further bolster their sense of resilience through peer discussions on how to assess challenges and identify solutions (Q17). Peers also offered each other much needed encouragement when taking the next steps to pursue goals, achieve new milestones, or choose an alternative path (Q18).

#### Clarification of values and goals

Participants mentioned that the curricular materials and exercises helped them identify and clarify their ideal career and life goals, while also bolstering their self-assurance about their personal values and choices (Q19). For some, this led to more intentional thought processes as participants pursued their ambitions at their home institutions. One participant shared how setting a goal during the program led to a key conversation with a senior leader at their institution, opening up a leadership opportunity that they were encouraged to pursue. They credited EMPOWER with helping them recognize and seize the opportunity with greater clarity and intention (Q20).

Participants noted that discussions with their peers provided the opportunity to examine and obtain feedback on career goals by sharing and comparing different experiences and viewpoints (Q21). They perceived the peer circle as allowing for the time and space to assess, question, or make decisions about personal values and leadership aspirations without pressure or judgment. For example, one participant valued the opportunity to openly discuss uncertainties about leadership, noting that others in the group shared similar questions and hesitations (Q22). A faculty advisor observed that one participant, after reflecting deeply, decided to focus on enhancing their current role rather than pursuing advancement for its own sake (Q23).

### Barriers to EMPOWER program participation, engagement, and commitment

**FIG. 2.**
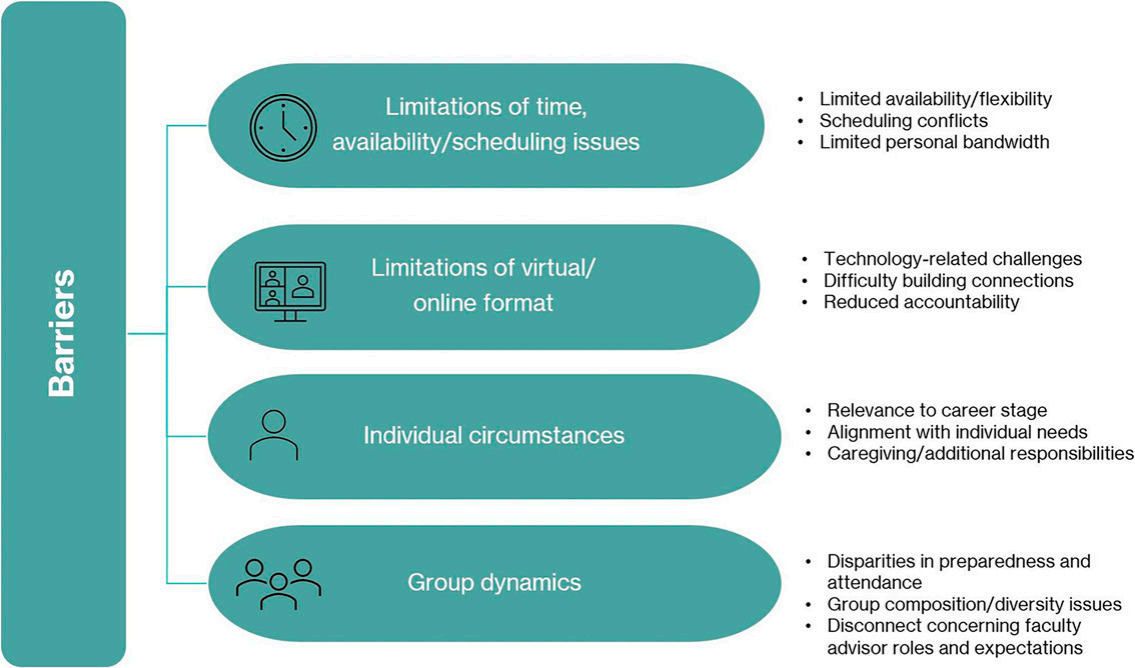
Perceived barriers to EMPOWER program participation, engagement, and commitment. These barriers were perceived to hinder full participation and engagement and, thereby, the ability to maximize and realize all potential benefits: (1) lack of time and limited availability/scheduling issues, (2) limitations of online/virtual format, (3) individual circumstances, and (4) group dynamics.

While experiences with the EMPOWER program were mostly positive, with many participants who perceived beneficial learning and professional development outcomes as described above, some participants reported being unable to fully participate at times and, therefore, did not feel that they were able to maximize or realize all the potential benefits. Perceived barriers to full program participation and engagement included a lack of time and limited availability/scheduling issues, limitations of an online/virtual format, individual circumstances, and idiosyncratic group dynamics (Fig. 2).

#### Lack of time and limited availability/scheduling issues

The most glaring barrier to program participation and engagement was a lack of adequate time to devote to readings, learning activities, and assignments, due to the various competing demands already placed upon participants with overlapping roles in clinical practice, research, teaching, and administrative duties within academic medicine. Those with more clinical practice, in particular, faced greater challenges with availability and scheduling flexibility. Participants noted that they simply did not have enough time to prepare between each monthly virtual meeting to be able to fully engage in meaningful discussions (Table 3; Q24), although they could still benefit from partially completing the preparatory work or listening to peers who had completed assignments (Q24, Q25). Some felt they needed more time during the peer circle meetings to engage in deeper discussions or bond with peers (Q26); however, they also acknowledged that it would be difficult to request participants to attend meetings for longer than 1 hour given the already limited flexibility and existing constraints in peers’ schedules.

Difficulty in agreeing upon a meeting time that fit everyone’s schedule in the peer circle was a frequently mentioned obstacle (Q27, Q28). Sometimes, there were conflicts between those who were open to scheduling meetings in the evenings or on weekends and others who preferred to meet only during work hours citing work–life balance reasons (Q27). Nevertheless, most participants tried their best to be flexible and accommodate the preferences of others in their peer circle.

#### Limitations of online/virtual format

Participants’ attitudes toward the use of technology or their level of tech-savviness sometimes hindered their engagement with the online learning platform or participation in the virtual meetings (Q29, Q30). Some observed that building bonds and relationships was difficult when interacting in an entirely virtual space (Q31). Not only could technical difficulties interrupt connections, but communication could feel awkward due to distractions or not being able to read facial expressions or body language (Q32). Participants also noticed that the level of accountability for completing assignments or attendance was lower in EMPOWER’s online environment compared with what they had generally experienced in the past when attending in-person meetings (Q33).

#### Individual circumstances

Participants’ family caregiving responsibilities were cited as an individual circumstance restricting already limited availability for scheduled meetings (Q34, Q35).

Another perceived barrier was that the program content, goals, or demands at times did not align with participants’ specific needs depending on their career stage. Participants felt that the curricular content was less relevant to them if they were more established in their careers and/or had different professional needs or concerns (Q36, Q37). Some noted that certain curricular content, such as the learning module focused on negotiation, would have been more helpful at a different, often earlier, point in their career trajectory (Q38).

#### Group dynamics

Participants mentioned various factors that could affect group dynamics in the peer circle, thereby making participation and engagement more challenging. Disparities in preparedness as well as attrition/absences were reported (Q39), often due to the barriers already described above. Additionally, some participants mentioned unbalanced or awkward group composition, such as a lack of diversity that limited the range of perspectives (Q40), or too many differences without enough commonality (Q41).

Some participants noted additional barriers concerning difficulties with group facilitation. There was, at times, a disconnect in terms of the faculty advisor’s comfort navigating the multiple roles needed to meet the various needs and expectations of their peer circle (Q42). These roles included facilitating discussions, ensuring balanced group interactions and meaningful integration of curricular content with real-life examples, serving as the initial group leader (with the goal of eventually enabling peer mentoring and co-leadership within the peer circle), and contributing as a senior mentor and advisor. When the faculty advisor was perceived as ineffective in one or more of these roles, it sometimes negatively affected the functioning of the peer circle (Q43, Q44, Q45).

### Recommendations to improve EMPOWER program quality and best practices

Participants shared detailed recommendations to enhance program participation, engagement, and commitment (Table 4). Key suggestions included more clearly communicating program goals, roles, and expectations through orientation and training, as well as customizing content to align with participants’ career stages, interests, and identities. Some suggested that it was important to showcase the program’s benefits to attract motivated candidates whose career goals closely align with the program. In addition, expanding the program beyond mid-career or research-focused participants was thought to increase inclusivity and impact.

**Table 4.** Recommendations to Improve EMPOWER Program Quality and Best Practices for Similar Programs^a^

Well-defined aims and expectations *Establish clear goals and program requirements upfront* Multiple (if possible) mandatory orientations/trainings should clearly outline/summarize program schedule, aims, content, and time demands for upcoming assignments so all participants have a chance to fully review important materials at the outset and can plan for participating and learning Curriculum guide should have time demands/timeline explicitly stated upfront Orientation/training should be expanded to include more meeting time to intentionally define and clarify peer circle roles, goals, and expectations (for both participants and faculty advisors) Ideally, programs should intentionally design exercises that can identify participants’ initial goals and expectations^a^
Tailored programming *Intentionally plan to customize the program design to closely align with individual circumstances/needs regarding career status, goals, and identity/life experiences* Program content and mentoring approaches might need to be adjusted for individuals at more advanced career stages Approach to curriculum content should consider the unique interests and needs of women leaders in academic medicine (*e.g.,* fertility, work–life issues, workplace dynamics and culture, research productivity, financial and budgetary knowledge) while incorporating a broad range of perspectives from all leaders and acknowledging the diversity in their experiences
Intentional recruitment *Messaging, outreach, and decision-making during the recruitment process should ensure that selected participants are highly motivated and best matched to program goals and parameters* Promote program benefits to attract candidates by highlighting the purposefully designed curriculum, the safe, supportive peer environment, and networking opportunities across institutions. Use alumni testimonials to showcase the program’s value. Candidates’ career and leadership goals should align closely with program goals (and associated content/agenda) Intentionally target ideal candidates at a key stage in their career when they are ready to take the next step and would benefit from the extra guidance/peer support Determine upfront if potential program participants are truly able to commit to program requirements and time demands May be beneficial to offer the program more broadly to maximize impact (as opposed to focusing only on the mid-career stage or research-oriented *per se*), though keeping it focused on women leaders may be preferred^a^ May be beneficial to ensure that individuals from underrepresented or minority groups are included in the recruitment/selection pool [Note: This could also be another reason to offer the program more broadly rather than relying solely on nominations or a restrictive application process (perhaps using a hybrid nomination/open application approach)]^a^
Efficient, purposeful use of time *Strategically plan the program schedule and format to maximize efficiency and productivity* Ideally, implement longer meeting times to fully process curricular material^a^ Ideally, enable more facilitators and peer circles to allow for smaller groups that can accomplish more, especially if sessions are limited to 1 hour^a^ Lengthen the program to allow for more meetings devoted to certain topics Strategically plan for the structure, pacing, and/or length of the more involved assignments
Labor-saving and minimization of mental load *Reduce participants’ time and effort spent on programmatic activities to encourage participation* Use email reminders to keep participants on track with assignments and help with time management and organization [Note: it is preferable for email reminders to contain attached content] Prioritize a user-friendly website/online learning platform Curriculum design/learning process should aim to reduce time burden, *e.g.,* incorporate audio books, short videos, or short summaries/snippets
Hands-on, interactive group learning experiences *Design and implement enhanced group learning experiences that promote applied learning and interaction* Balance didactic content and interactive group reflections Include more hands-on group activities (*e.g.,* practicing negotiation, self-promotion) to develop practical skills and strengthen group connections Create peer mentoring opportunities within sessions to encourage participants to share challenges and offer advice and support Consider including a dedicated session focused on salient topics that extend beyond the intended curriculum to address specific interests and needs brought up organically during the peer circle discussions
Online community-building *Prioritize the establishment of participants’ connections and engagement with peers so they have a safe space in the virtual environment to share, collaborate, learn from, and support each other* Consider establishing a hybrid program to allow for some in-person meetings (*e.g.,* an in-person meeting at the beginning of the program to better establish bonds upfront and/or a meeting at the end of the program to solidify connection to a larger initiative)^a^ Provide techniques for effective online/virtual communication and interaction during orientation/training Consider using written bios to introduce participants to each other at the beginning of the program^a^ Allow sufficient time to establish bonding upfront (*e.g.,* longer meeting times, more frequent meetings initially) Allow for continued bonding and interaction throughout the program in flexible and alternative ways (*e.g.,* more unstructured time for establishing rapport during each meeting or time to connect in-between sessions; additional options for interacting with peers outside of the monthly meeting) Intentionally group peer circles so they have enough in common (*e.g.,* similar career stages, goals, work–life schedule preferences, and areas for growth/improvement), but with some diversity of perspective When possible, facilitate additional one-on-one meetings to build more connections^a^ Facilitate ongoing meetings for EMPOWER alumni to re-establish connections
Support the role of faculty advisor *Ensure purposeful, strategic involvement of the faculty advisor and provide needed support* Ensure that faculty advisors have relevant experience, understand participants’ challenges, and are willing to share their insights [Note: they should have a genuine belief in the value of leadership programs and peer support] Provide facilitators with comprehensive guidelines, orientation/training, and resources Consider incorporating multiple, rotating presenters to share different perspectives and expertise^a^
Encourage accountability without discouragement *Design program with built-in mechanisms for regular feedback and check-ins to keep participants engaged and on track* Provide feedback on assignments to encourage accountability and commitment to do the work Where feasible, build more ways for participants to interact with one another, discuss, and obtain feedback, even outside sessions, in small but regular amounts^a^ Utilize check-ins, regular reevaluations, or one-on-one sessions to ensure participants stay engaged and on track Consider assigning a participant to lead a meeting in terms of curricular content and rotating that responsibility among the group^a^ Celebrate participants who come prepared to reinforce accountability, while creating opportunities for participants who may not be fully prepared to engage meaningfully in discussions Ideally, foster accountability and institutional engagement by integrating mentoring plans and protected time for career development, participant speeches, and/or feedback for institutions at the end of the program^a^ Encourage faculty advisors to encourage participant participation and attendance
Recognition and visibility *Messaging, events, and institutional outreach/involvement should emphasize the program’s prestige and include opportunities for participant recognition/visibility to promote commitment and enthusiasm* Highlight the program’s prestige and create outlets for recognition/visibility; for example, accolades on CV; sponsorship/institutional support from a Dean or Chair, nomination (or hybrid nomination/open application process if program is offered more broadly), and/or connections to larger networks like ELAM Consider a formal, national, in-person meeting for all participants to highlight their involvement in a broader initiative^a^

a We recognize that different institutions may have varying resources, rules, and constraints that will affect their desire or ability to implement some of these recommendations. These site-specific variations are to be expected and carefully considered as stakeholders seek to develop programs that optimally meet their institution’s goals and needs. Recommendations marked with an asterisk are optional and can be adapted based on idiosyncratic circumstances and the specific needs of each institution hosting the program.

ELAM, Executive Leadership in Academic Medicine.

Intentionally optimized program structure and efficiency were identified as critical, with recommendations to allocate more time to key topics and assignments while reducing mental burdens by streamlining access to material and email reminders. To improve learning experiences and engagement, participants suggested balancing didactic content with hands-on activities with practical, actionable objectives, integrating peer mentoring opportunities, and dedicating a session for participant-selected discussion topics. To overcome virtual format limitations, incorporating hybrid options with in-person meetings, shared written bios with photos to facilitate introductions and strengthen recognition, alternative communication venues, such as discussion boards, and additional one-on-one sessions could strengthen bonding and networking.

Effective faculty advisors were described as those with a strong belief in the program’s value for fostering leadership in academic medicine, especially those who have a comprehensive understanding of faculty development and a broad range of experiences that allow them to effectively address the challenges experienced by program participants. Providing faculty advisors with training, clear guidelines, and opportunities to incorporate rotating experts could enrich the quality of facilitation. Accountability and engagement can be reinforced through regular feedback, rotating leadership roles within the peer circles, one-on-one check-ins, and faculty advisors recognizing accomplishments while supporting those facing challenges.

Finally, increasing program visibility through institutional support, nomination processes (or a hybrid nomination/open application model for inclusivity), connections with established networks such as ELAM®, and a formal national meeting could boost engagement, opportunities for more widespread professional recognition, and a sense of accomplishment at being part of a broader initiative.

In sum, many program participants and their faculty advisors reported positive experiences and benefits from EMPOWER. In particular, the peer circle appeared to play an important role in reinforcing the learning of curricular content in addition to presenting new, outside perspectives. It also provided needed social support, encouragement, and guidance for how to practically apply skills in pursuit of career goals. Lastly, while some observed barriers prevented them from fully maximizing benefits, many participants recounted rich experiences and impressions. Detailed recommendations were made to improve quality and best practices, helping to reduce barriers and amplify benefits in future iterations of the program. These recommendations are valuable but should be adapted based on institutional goals, available resources, and participant needs to ensure successful and effective implementation.

## Discussion

In this qualitative study, we investigated the factors impacting program feasibility, optimization, and scalability through in-depth interviews with EMPOWER participants and focus groups with their faculty advisors. Qualitative analysis of their recounted lived experiences and observations highlighted the peer circle’s important role in enhancing leadership learning and engendering positive professional development outcomes. It also identified the barriers impacting program participation and engagement, informing best practices and opportunities to improve program quality.

The benefits described by program participants and their faculty advisors reinforce the value of women’s leadership development programs, especially those that include a peer mentoring component.^10–12^ Such peer interactions may be especially useful in providing participants with external information, resources, and feedback that they otherwise would not be able to access at their local institutions due to workplace environment or politics. Participation in a national program with peers from different institutions who share similar experiences may also serve to provide emotional support and validation for isolated or marginalized women faculty while ensuring safety and anonymity. Our findings align with other studies on cross-institutional peer mentoring programs,^28,29^ which show that these programs facilitate the exchange of external strategies and practices and helped participants feel safer and more comfortable sharing challenges while avoiding institutional politics and confidentiality concerns. Furthermore, the networks formed through the program can potentially evolve into peer sponsorship when sustained over time. The experiences, observations, and recommendations voiced by program participants and their faculty advisors are consistent with what other scholars have previously identified as barriers to online learning in medical education and proposed best practices for ensuring quality and efficiency.^30,31^ Participants echoed common barriers, such as technological challenges and engagement difficulties in online learning environments. This study also reflects barriers noted in literature on virtual mentorship and peer mentoring, particularly the challenges of cultivating meaningful relationships and maintaining long-term commitment in a virtual setting.^11,32^ Programs designed to support women’s advancement in academic medicine should reduce, to the greatest extent possible, barriers related to labor and mental load. Evidence suggests that a lack of time and flexibility, which can be further exacerbated by parental or caregiving responsibilities, is especially salient and burdensome for women.^33^ These challenges can limit their ability to fully engage in leadership development opportunities to advance in their careers.

That some participants desired more clearly communicated goals, roles, expectations, and training of faculty advisors speaks to the need for extensive preparation and oversight of such initiatives and the tradeoffs of a purely virtual program. Of note, these concerns were articulated even in the context of a program that had been developed *via* a collaborative expert team over multiple years and with the participation of numerous experts in leadership training.^14^ We intentionally developed a strictly virtual program that limited requirements for synchronous participation to maximize flexibility of scheduling in a cohort known to face many competing time pressures,^33^but this approach did involve tradeoffs. Specifically, participants who did not review the extensive materials available to them might have been better served by a program that required a greater time commitment and perhaps some degree of in-person interaction that might have motivated greater engagement. Future programs that desire to pursue a purely virtual format should consider incorporating additional mandatory orientations and trainings to ensure all participants review important content at the outset, consistent use of reiteration/reminders to maximize retention, and dedicated time during meetings to further digest the information about the goals and their roles with their peer circle.

Given the need for scalable programs that can reach emerging women leaders on a broader scale, particularly at a time when academic institutions are facing financial constraints on the ability to support faculty travel, approaches that can reduce the barriers inherent to virtual programming should be further explored and implemented. The virtual format of EMPOWER offers participants flexibility. Evidence suggests that virtual mentorship programs^34–36^ can increase accessibility, convenience, and cost-effectiveness, all of which are crucial for women in academic medicine who face competing demands, such as caregiving responsibilities or challenges with traveling frequently for in-person meetings. Also, virtual platforms may further equity by enabling quieter voices to be heard, through typing into the chat or virtual hand raises,^36^ for example. Interestingly, several participants mentioned that a hybrid format in particular might be preferable and potentially more effective, which is consistent with prior qualitative findings on the preferences of medical faculty.^37^ This suggests that while convenience and accessibility is important for busy faculty clinicians, it remains imperative that programs maximize to the greatest extent possible meaningful in-person engagement and longer-term personal connections. Hybrid programs that enhance virtual meetings and an online curriculum by strategically incorporating limited but impactful in-person experiences may provide the best of both worlds. Future structured in-person EMPOWER components could include an initial retreat for participants to meet and outline goals. This could be followed by a midpoint session to strengthen peer interactions and discuss both challenges and opportunities among the group. Finally, a closing symposium to highlight accomplishments and discuss future virtual and in-person interactions could be incorporated into the program. These interactions would strengthen peer interactions and foster a deeper sense of community that could be continued both in person and virtually. Combining virtual sessions with in-person interactions might optimally facilitate enduring peer relationships and foster new connections.

We acknowledge that the in-person interactions also carry with them a financial burden superimposed upon a time burden.^34–36^ In-person programs carry costs not only for organizers but also for participants. They necessitate travel, time off work, and making the required accommodations at home, including securing childcare, which may be particularly difficult for those at earlier stages in their career, as well as care for adult kin, which may be difficult across many age groups. Moreover, geographic and mobility constraints may further limit accessibility and equity. In under-resourced institutions, these burdens are likely to be even greater, posing an additional obstacle to select participants. The benefits of in-person interactions therefore must be weighed against the cost and accessibility for participants. Ultimately, hybrid models should be designed to promote equity among participants and offer realistic opportunities for participant engagement. By enhancing access and flexibility while preserving the most crucial aspects of in-person communication and networking, hybrid programming can support a more inclusive and effective approach to leadership development if harmonious integration and ideal balance are achieved. This would help ensure that women have the opportunity to thrive in academic medicine despite the challenges they face.

Notably, some participants reported less engagement with the materials or their peer circle if they perceived that the curriculum content, learning goals, or conversation topics were not directly relevant to their career stage or professional interests. Given the limited time available for career development, especially in the context of virtual or hybrid formats, the importance of tailored programming to maximize results while reducing the extra burden of superfluous content seems especially important to consider. To enhance meaning and impact, program designers could consider incorporating goal customization, individualized learning paths, and groups carefully assembled to heighten peer compatibility for more dynamic interactions. To better support such individual needs in navigating career progression and transitions, program planners should seek to incorporate established frameworks, such as the Faculty Career Self-Management Model,^38^ or milestones designed for faculty development in academic medicine.^39^ These frameworks enable participants to anticipate and reflect on their stage-specific needs, so that they can align program and mentoring opportunities with personal goals as well as seek out more compatible peer groupings and support networks. The recommendations that we present here to improve the EMPOWER program and others like it are informed by the rich experiences of our study participants—highly accomplished women medical faculty, many of whom have limited bandwidth given their leadership roles and responsibilities. These recommendations offer a simple and adaptable guide for administrators of similar virtual programs for women in academic medicine, helping them proactively address barriers prior to implementation to maximize participation, engagement, and commitment. It should be noted, however, that women-only leadership programs do not currently exist in the same way as when the research team initially conducted this study due to recent policy developments and the continually evolving landscape in academic medicine. Nevertheless, these rich qualitative accounts are germane to leadership development programs more broadly.

Moreover, the recommendations herein can serve as a highly informative guide as administrators seek to reimagine how they will develop more widely inclusive and accessible programs going forward that will benefit the advancement of all promising leaders in their academic medical careers.^40^ Women’s leadership programs are under increased scrutiny secondary to the legal and political initiatives aimed at gender-specific initiatives,^40,41^ not necessarily because of waning interest or evidence of ineffectiveness. These pressures underscore the importance of creating programs that offer flexibility, with the ability to shift with institutional directives and changes in society and social norms. The EMPOWER program structure and content are highly adaptable; thus, it can be readily modified in the face of the rapidly shifting landscape of gender-aware leadership development programming toward broad inclusion and accessibility.

However, as championed in a recent statement from ELAM,^41^ change in ability to host gender-specific programming does not mean that a program’s mission must completely change, although the strategies to work toward that mission may have to be altered. Like ELAM, programs can continue to challenge and reform the institutional structures that hinder women and others from assuming influential positions.^41^ With gender-inclusive programs, total reach can be broadened, furthering dialogue across a wide range of lived experiences. This approach can augment impact and galvanize a wider community of leaders to aim for transformative change.^41^ These programs must continue to be evidence-based as they evolve, while continuing to acknowledge and address the gaps that exist in academia among different gender and racial or ethnic groups.

Furthermore, it is important to carefully consider the risks and implications of expanding access to programs versus losing the safety and solidarity that many women valued in gender-specific spaces. In gender-specific spaces, program participants may feel more comfortable discussing sensitive challenges such as bias and discrimination^42^ and may benefit from connecting with mentors and peers with similar lived experiences.^43^ Gender-specific programming can also offer targeted skill-building particularly for areas in which gender disparities continue to exist, such as negotiation^44,45^ and sponsorship.^21^ Indeed, the loss or dilution of gender-specific spaces poses several possible downsides, including blunted group bonding dynamics resulting from a reluctance to fully share experiences in perceived unsafe spaces, weakened networking opportunities because some may find it more difficult to cultivate peer connections in mixed settings, and pressure to conform to typical “male” leadership norms^46^ rather than exploring diverse approaches to leadership.

In light of this, identity-centered cohorts might serve as a middle ground. Identities are complex, and gender is only one of the identities any given person may bring to the conversation. Future programs could seek to invite participants to indicate whether they would prefer to meet in a mixed identities group or a shared identity group. Program administrators can inquire as to what identities are important to participants, whether it be gender, race/ethnicity, medical specialty, region, parental status, career stage, or another factor, and try to organize those shared identity meetings, if possible. If such shared identity spaces are not feasible, programs should foster environments that support and encourage individual participants in voicing issues associated with their identities as they would like to raise them, both in the group setting and to the facilitator or faculty advisor.

This study has potential limitations. Participants who accepted the interview invitation may have been more likely to have been engaged or to hold more favorable views of the program, potentially leading to an overrepresentation of positive experiences. However, we deliberately made efforts to include some participants who were ultimately unable to complete or fully engage in the program to gather data on their unique perspectives and experiences. Since interviews were conducted either at the program’s midpoint or a few months after its completion, this qualitative study did not capture longer-term impacts or reflections on the program. Additionally, the scope of our analysis did not assess for thematic differences between the midpoint and end of the program.

A particular strength of this study is its adherence to rigorous qualitative methods. A purposeful sampling strategy^15–16^ was used to maximize the diversity and range of viewpoints gathered, and adherence to thematic saturation^19,20^ ensured that the data collected reflected sufficient depth and variation. Two forms of triangulation^47^ were employed to strengthen the validity of the study findings. First, the analytical team varied in training as well as gender and racial identities, which enhanced investigator triangulation and diversity of perspectives. Second, data source triangulation was achieved by gathering both individual interviews with program participants and focus groups with their faculty advisors, which provided complementary accounts that were also inherently different in nature. Another strength of this study is that the rich, detailed information concerning program quality improvement and best practices is based on actual lived experiences and insights of those who participated, ensuring the recommendations are participant-centered and more likely to lead to actionable and meaningful changes that enhance the program for future participants.

## Conclusions

Overall, the findings of this qualitative study suggest that the EMPOWER program is a promising, scalable intervention. It can inform the development of future programming that seeks to be inclusive on a broad scale, particularly regarding the gender-aware content that is crucial knowledge for all leaders in academic medicine. Program administrators should thoughtfully consider how to minimize barriers to participation and engagement, especially those associated with limited time and flexibility. Moreover, they should implement best practices for optimizing and improving program quality to enhance individual and group experiences through purposeful planning, tailored programming, intentional recruitment, and efficient implementation.

## Ethical Approval

The University of Michigan Institutional Review Board approved this study as part of a randomized trial to evaluate its impact (HUM00185660).

## Authors’ Contributions

R.D.J.: Project administration, data collection, analysis/interpretation, writing—original draft, and writing—review and editing. Y.-J.L.: Data collection, analysis/interpretation, writing—original draft, and writing—review and editing. J.D.T. and A.M.B.: Analysis/interpretation and writing—review and editing. N.D.S.: conceptualization, interpretation, and writing—review and editing. C.M.C.: Interpretation and writing—review and editing. K.S.: conceptualization, interpretation, and writing—review and editing. K.C.P.: interpretation and writing—review and editing. E.A.K., E.L.F., A.J.S., I.H.S., and P.A.U.: Conceptualization, interpretation, and writing—review and editing. R.J.: Conceptualization, interpretation, funding acquisition, supervision, and writing—review and editing.

## Abbreviations

ELAM®: The Hedwig van Ameringen Executive Leadership in Academic Medicine program
EMPOWER: Engaging Peer Mentors for Opportunity, Well-Being, and Equity Realization
FTE: full-time equivalent
NIH RePORTER: National Institutes of Health Research Portfolio Online Reporting Tools Expenditures and Results
URM: underrepresented race and ethnicity in medicine
